# Reirradiation of recurrent salivary gland malignancies with fractionated stereotactic body radiation therapy

**DOI:** 10.1007/s13566-012-0010-6

**Published:** 2012-02-19

**Authors:** Sana D. Karam, James W. Snider, Hongkun Wang, Margaux Wooster, Christopher Lominska, John Deeken, Kenneth Newkirk, Bruce Davidson, K. William Harter

**Affiliations:** 1 Department of Hematology/Oncology, Georgetown University Hospital, Washington, DC USA; 2 Department of Otolaryngology, Georgetown University Hospital, Washington, DC USA; 3 Department of Radiation Medicine, Georgetown University Hospital, 3800 Reservoir Rd., NW, Washington, DC 20007 USA; 4 Department of Biostatistics, Bioinformatics, and Biomathematics, Georgetown University Hospital, Washington, DC USA; 5 Department of Radiation Oncology, The University of Kansas, Kansas City, KS USA

**Keywords:** Salivary malignancies, Reirradiation, Stereotactic radiosurgery, Toxicity

## Abstract

**Purpose:**

The purpose of this study was to review a single-institution experience with the reirradiation of recurrent salivary gland tumors using fractionated stereotactic radiosurgery (SBRT).

**Methods:**

Between 2003 and 2011, 18 patients diagnosed with recurrent, previously irradiated, salivary gland carcinomas were treated with SBRT reirradiation. Median age was 68 for all patients with most tumors being of major salivary gland origin. Most patients did not undergo surgical resection, and among those that did, all had positive margins. Only seven patients received chemotherapy, and the median SBRT dose was 30 Gy given in five fractions with a median cumulative dose of 91.1 Gy.

**Results:**

The median overall survival (OS), progression-free survival (PFS), and local control (LRC) were 11.5, 3.5, and 5.5 months, respectively. The 2-year OS, PFS, and LRC rates were 39%, 24%, and 53%, respectively. Statistical analysis identified presence of gross disease and interval to reirradiation as negative predictors of survival outcomes on both univariate and multivariate analyses (*p* < 0.05). On multivariate analysis, tumor volume was a negative predictor of survival outcomes (*p* < 0.05). Long-term toxicity analysis revealed four patients in the reirradiated group with soft tissue necrosis, which correlated with the cumulative dose (*p* = 0.01).

**Conclusion:**

Our data suggest that SBRT is a reasonable treatment option for reirradiation of salivary gland tumors, but further studies are warranted.

## Introduction

Salivary gland cancers are rare, comprising only 3–5% of all head and neck cancers, with a diverse spectrum of histologic subtypes and natural history [1]. Multiple retrospective studies have shown that in high-grade tumors, advanced stage (T3/4), and/or inadequately excised tumors, adjuvant RT is superior to surgery alone [2–5]. For patients with recurrent salivary gland tumors, the prognosis is poor, largely because effective salvage treatment is often limited by the infiltrative pattern of local growth as well as the proximity of the recurrent tumor to critical structures [6]. Since a large proportion of these patients have previously received radiation therapy, the potential for severe complications often prohibits the delivery of additional radiation using standard external beam techniques. Salvage surgery with intraoperative radiation treatment [6], chemoirradiation with photon beams [7], and reirradiation with neutron beam therapy [8] are strategies that have been utilized with limited success.

Stereotactic radiosurgery (SBRT) represents an appealing option for the management of salivary gland tumors either as a means of dose escalation or for reirradiation of recurrent tumors. SBRT uses multiple convergent beams with various targeting techniques to deliver highly conformal treatment accurately. Gamma knife-based SBRT technologies, which require external frame-based fixation devices, have been previously used in the retreatment of recurrent salivary gland tumors involving the skull base [9, 10]. Local control rates were reported at 100% and 75% [9, 10]. The CyberKnife SBRT system (Accuray, Inc., Sunnyvale, CA) allows conformal treatment of sites throughout the head and neck region. This system uses real-time image guidance for targeting without rigid external fixation [11]. Multiple treatment sessions can be used, potentially reducing late normal tissue toxicity via dose fractionation. We report our institutional experience with fractionated SBRT irradiation of salivary gland cancers, addressing feasibility, safety, and outcomes.

## Patients and methods

### Patients

Eligible patients were diagnosed with recurrent malignant salivary gland tumors and were treated with definitive SBRT reirradiation either as a primary or adjuvant modality at the Georgetown University Hospital between September 2003 and March 2011. All patients had histologically proven disease. Details of the previous radiation therapy were available for all patients with prior history of salivary gland irradiation. The data were reviewed under an institutional review board-approved retrospective protocol. A total of 22 patients who met entry criteria were identified. Four patients were lost to follow-up and were excluded from the study. The final group consisted of 18 patients with prior salivary gland tumor irradiation whose recurrent tumors either were unresectable or had gross residual disease or positive margins postoperatively. Two of the 18 patients received SBRT as part of their primary treatment, and a second course of SBRT for their local recurrence was included in local control and progression-free survival analysis but excluded from the overall survival analysis. Before the treatment, patients' cases were reviewed at the multidisciplinary head and neck tumor board. When feasible, patients underwent surgical resection. Other than determining presence or absence of gross disease, no patient selection was performed. Radiosensitizing concomitant chemotherapy was administered at the discretion of the treating medical oncologist. The most common chemotherapy was carboplatin administered in three cycles. The first cycle was given 1 week prior to the initiation of the radiation treatment followed by another cycle administered concurrently with the treatment. The third cycle was given the week following radiation treatment.

### Fractionated stereotactic body radiation treatment

The CyberKnife SBRT system (Accuray, Inc., Sunnyvale, CA) uses a 6-MV X-band linear accelerator mounted on a fully articulated robotic arm. During treatment, two orthogonally positioned x-ray detectors provide real-time imaging of bony anatomy allowing for intrafraction movement correction. Treatment was generally administered on an outpatient basis with each treatment lasting approximately 45–90 min. Most of the patients received their treatments over the course of 7 days, consecutive with the exception of holidays or weekends.

Patients were immobilized in the supine position with an Aquaplast facemask (WRF/Aquaplast Corp., Wyckoff, NJ). All patients underwent a treatment planning computed tomography (CT) scan, fused with a fluorodeoxyglucose-positron emission tomography (FDG-PET) scan with 1.0-mm-thick slices. In all cases, magnetic resonance imaging (MRI) scans with VIBE sequence and 1-mm slices were also used in planning. The MRI and PET images were then fused with that of the simulation CT scan for treatment planning. The gross tumor volume (GTV) was reconstructed based on the information obtained from both the PET and the MRI together. The PET-GTV was usually contoured to the halo. The sum of both the PET-contoured volume and the MRI-contoured volume defined the GTV. Once the GTV was contoured, an expansion was done ranging from 2 to 10 mm depending on the pathologic margin status and proximity to critical structures to define the clinical tumor volume. No additional margin was added for the planning target volume (PTV). In postoperative cases, the preoperative MRI was also fused with the postoperative PET and MRI, and the sum of all volumes defined the postoperative PTV. In seven cases, adjacent soft tissue and immediate draining lymph nodes were targeted as a separate PTV. In patients who underwent surgical resection, the PTV encompassed the entire surgical bed when feasible. The median reirradiation dose was 30 Gy but varied between 21 and 40 Gy depending on prior dose, interval to reirradiation, and tumor burden. Similarly, the median dose per fraction was 5 Gy and varied between 2 and 7 Gy. Of these fractionation schemes, the most frequently used was 30 Gy in five fractions (seven patients). For neck irradiation, ipsilateral neck irradiation was given as five fractions, 7 Gy each for a total dose of 35 Gy.

Inverse planning was used to determine the dose to the target volume while minimizing the dose to normal tissue. All planning was completed within 1 week of imaging, and typically, patients initiated treatment in 2–3 weeks after imaging depending on chemotherapy coordination. Treatment was generally completed within 7 days of initiation, consecutive with the exception of holidays or weekends.

### Post-treatment follow-up

Patients typically underwent a post-treatment surveillance with an MRI scan 3 months after the completion of SBRT and then every 6 months thereafter (with a FDG-PET/CT scan and MRI). Radiographic imaging was done on follow-up to monitor disease recurrence locally, regionally, or distantly. For those with gross disease, PET and MRI were used to monitor “response” to treatment as well as regional and distant metastases. For those without evidence of gross disease on treatment initiation, radiographic evaluation was done to monitor disease recurrence locally, regionally, or distantly. Clinical examination was conducted at the same interval, with biopsy as indicated. Acute and late toxicity were graded using the Radiation Therapy Oncology Group (RTOG) scoring criteria.

### Statistical analysis

Progression-free survival (PFS) was defined as the time from the first day of SBRT treatment to local/distant failure or last follow-up in living patients without evidence of recurrence/progression. Locoregional control (LRC) was defined similarly except that death and distant failure were not considered events. Patients were censored at the time of death. Overall survival (OS) was the time from SBRT treatment until death or last follow-up. PFS and LRC were evaluated among the subset of patients treated definitively. OS was evaluated among the subset treated definitively and for all patients. Interpretation of available FDG-PET/CT, MRI, and CT scans with correlative clinical examinations was used to assess for response of the treated lesion 2–3 months after SBRT. Complete response was defined as no evidence of disease in the treatment volume by both radiographic and direct clinical examination. No response was defined as absence of marked change or increase in the treated lesion. Partial response was defined as not meeting the criteria for complete response or no response. Log rank tests and Cox regression models were used to evaluate the association between clinical factors and each survival outcome. The independent variables considered were surgery (yes, no), nodal status (yes, no), presence or absence of gross disease (yes, no), SBRT dose (<35 Gy, ≥35 Gy), interval to reirradiation (<12 months, > 12 months), grade (low, medium, high), concurrent chemotherapy (yes, no), nodal stage per the AJCC staging system, age in years, size in centimeters, SBRT dose in grays, and cumulative dose in grays. The presence of positive margins was coded as absence of gross disease since most patients had one or the other. Multivariate analysis was conducted for selected factors while adjusting for age or presence of gross disease in the Cox model. Kaplan–Meier plots are presented for selected significant factors. For long-term toxicity analyses, dysphagia (present, absent), fibrosis (present absent), and soft tissue or bone radionecrosis (present, absent) were the dependent variables. Correlation of toxicity variables with SBRT dose was done using logistic regression. The correlation between the presence or absence of diabetes and development of soft tissue necrosis was examined using chi-square test. Analyses were performed in SAS version 9.2 (SAS Institute Inc., Cary, NC).

## Results

### Patient characteristics and disease presentation

Baseline patient and disease characteristics are listed in Table 1. Median patient age at the time of treatment was 68, 13 patients were males, and the majority of the tumors were of the parotid glands. The most common histopathologic subtype was squamous cell carcinoma while adenocystic, acinic, and adenocarcinoma were equal (Table 1). Although the majority of the patients [11] had N0 disease, five patients had N2 disease.

**Table 1:** Patient characteristics

	Reirradiation
	*n* = 18
Age at diagnosis (years)	
Median	68
IQR	60–81
Gender	
Male	13 (72)
Female	5 (28)
Primary site	
Major salivary	12 (67)
Minor salivary	3 (16)
Other	3 (16)
Histology	
Adenoid cystic	2 (11)
Mucoepidermoid	1 (6)
Adenocarcinoma	2 (11)
Acinic	2 (11)
Squamous	7 (39)
Other	4 (22)
Tumor size (T stage)	
Recurrent	18 (100)
N stage	
0	11 (61)
1	2 (11)
2	5 (28)

### Treatment characteristics

Treatment characteristics are presented in Table 2. The majority of [10] patients did not undergo surgery (seven did not, three attempted). Among the eight patients that did, all had positive margins. Of the seven patients with nodal disease, four underwent ipsilateral neck dissection, and three did not. Four of the patients (one definitive and three adjuvant) received ipsilateral neck irradiation. Adverse pathologic included three (17%) patients with perineural involvement, four (22%) had facial nerve involvement, and two (11%) with lymphovascular involvement or extracapsular extension (Table 2). The majority of the patients [11] did not receive chemotherapy (Table 2). Treatment doses were discussed under the “Fractionated stereotactic body radiation treatment” section above and are also shown in Table 2.

**Table 2:** Treatment characteristics

	Reirradiation
	*n* = 18
Surgery	
Yes	8 (44)
No	10 (56)
Gross disease	
Gross (no surgery)	10 (56)
No gross	8 (44)
Margin for surgery patients (*n* = 8)	
Positive	8 (100)
Negative	0 (0)
Perineural invasion	3 (17)
Lymphovascular invasion	2 (11)
Major nerve involvement	4 (22)
Extracapsular extension	2 (11)
Chemotherapy	
Yes	7 (39)
No	11 (61)
Neck irradiation	
Yes	7 (39)
No	11 (61)
Median SBRT dose	30
Range	21–40
Median SBRT dose/fraction	5
Range	2–7
Median total dose (Gy)	91.1
Range (Gy)	62.4–121

### Clinical outcomes and prognostic factors

**Fig. 1:**
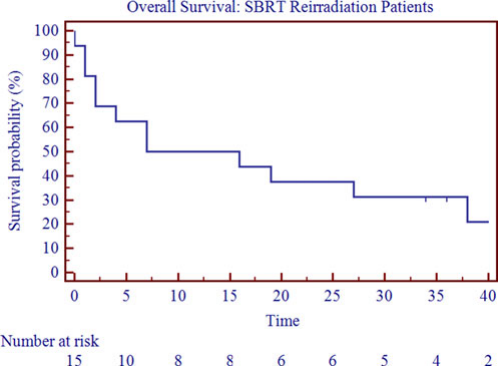
Overall survival for SBRT reirradiation patients (*n* = 16)

**Fig. 2:**
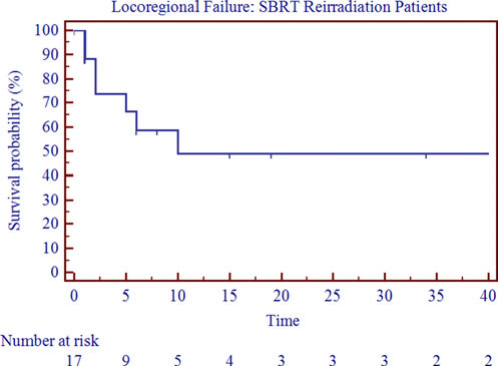
Locoregional control for SBRT reirradiation patients (*n* = 18)

**Fig. 3:**
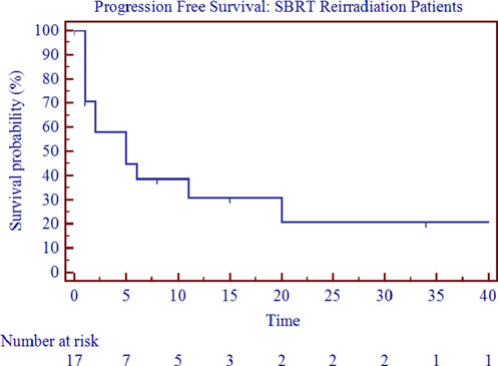
Progression-free survival for SBRT reirradiation patients (*n* = 18)

**Fig. 4:**
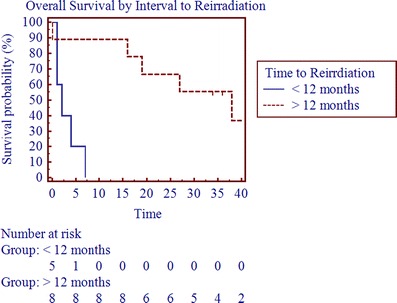
Overall survival by interval to reirradiation

With a mean follow-up of 20 months (range, 0–88 months; median follow-up, 12 months), the median OS, PFS, and LRC were 11.5, 3.5, and 5.5 months (Table 3; Figs. 1, 2, and 3). The 2-year OS, PFS, and LRC rates were 39%, 24%, and 53%, respectively (Table 3). Crude survival outcomes are also shown in Table 3. Forty-four percent failed locoregionally, 44% failed distantly, and the cancer-specific mortality was 50% (Table 3). Univariate analysis revealed that presence of gross disease and the interval to reirradiation were negatively correlated with overall survival (*p* < 0.05, Table 4 and Fig. 4). On multivariate analysis after adjusting for age, interval to reirradiation (*p* = 0.0197; HR, 0.0650, 95% CI 0.0066–0.6389), gross disease (*p* = 0.0446; HR, 4.5354, 95% CI 1.0446 to 19.69), and tumor volume at the time of reirradiation (*p* = 0.0412; HR, 1.0034, 95% CI 1.0022 to 1.0066) were significant predictors of OS. On multivariate analysis, tumor volume at the time of reirradiation was also statistically correlated with progression-free survival (0.0329; HR, 1.0038, 95% CI 1.0003 to 1.0073). A statistically significant correlation was seen between SBRT dose and LRC with doses above 35 Gy yielding better LRC rates (*p* < 0.05). However, it is important to note that the analyses is solely based on four events, since only four patients were treated with doses greater than 35 Gy, which limits the ability to draw any definitive conclusions.

**Table 3:** Crude survival outcomes

	Reirradiation
	*n* = 18
Mean follow-up (months)	19
Range	2–88
Local failure	8/18
Percent (%)	44
Distant failure	8/18
Percent (%)	44
Any progression	13/18
Percent (%)	72
Cancer-specific mortality	9/18
Percent (%)	50
Overall mortality	16/18
Percent (%)	89
2-year actuarial	0.39
Overall survival	(0.16–0.61)
2-year actuarial	0.53
Locoregional control	(0.28–0.79)
2-year actuarial	0.24
Progression-free survival	(0.01–0.46)

**Table 4:** Univariate analysis. Hazard ratio analysis of survival outcomes for prognostic factors. Abbreviations Overall survival (OS), progression-free survival (PFS), and locoregional control (LRC), *p*-value (*p*), hazard ratio (HR), 95% confidence interval (95% CI)

		OS	LC	PFS
Surgery	*p*	0.3806	0.3458	0.8091
	HR	0.5648	2.2152	1.1642
	(95% CI)	(0.1584–2.0138)	(0.427–11.477)	(0.3413–3.9711)
Gross disease	*p*	0.0270	0.4709	0.6299
	HR	0.198	0.5460	1.3462
	(95% CI)	(0.047–0.832)	0.1063 to 2.8054	(0.4043–4.4822)
Age	*p*	0.1192	0.1418	0.3598
	HR	1.039	1 .044	1.020
	(95% CI)	(0.990–1.091)	(0.986–1.107)	(0.978–1.065)
Nodal status	*p*	0.6357	0.3707	0.2565
	HR	1.159	1.463	1.516
	(95% CI)	(0.630–2.130)	(0.636–3.363)	(0.739–3.113)
Reirradiation interval	*p*	0.0159	0.8525	0.1669
	HR	0.0659	1.2421	0.3571
	(95% CI)	(0.0073–0.5947)	(0.1279–12.0619)	(0.0835–1.5267)

### Toxicity

Six patients experienced RTOG grade 1 to 3 acute toxicity, including mucositis, dermatitis, and nausea. These complications were generally transient and resolved with conservative management. One patient (3%) experienced RTOG grade 4–5 acute toxicity as he developed aspiration pneumonia within a month from initiation of treatment and died as a result. Two patients (11%) developed fibrosis. Both had surgical resection with neck dissection in addition to the radiation treatment. Six patients (33%) experienced dysphagia. None, however, required a feeding tube. Severe late radiation-induced toxicity was limited to soft tissue necrosis (STN) seen in four patients (22%). All of these patients were salvaged by surgical reconstruction. There was a statistically significant correlation between SBRT dose, as well as cumulative dose, and the development of STN (*p* = 0.004, *p* < 0.001). Plots of the STN vs. cumulative dose revealed that the risk of STN arises near 90 Gy, but as there are only four observations of STN in the group, a definitive conclusion cannot be made. There also appears to be a statistically significant correlation between diabetes and development of soft tissue necrosis (*p* = 0.025) using chi-square test.

## Discussion

The present study described our recent experience with the reirradiation of salivary gland cancer patients using fractionated stereotactic body radiosurgery. Our work focused on a small subset of this rare disease, those that have recurred in a previously irradiated site. Published reports of definitive radiotherapy for recurrent salivary cancer demonstrate generally poor locoregional control rates [6–8, 10]. In previously irradiated tissue, the proximity to critical structures often prohibits the delivery of additional radiation using standard external beam techniques. Fractionated SBRT allows for delivery of highly conformal treatment of targets that are in close proximity to critical structures. Fractionation has been hypothesized to improve the therapeutic ratio, thereby reducing the risk of late complications potentially associated with a large single dose [11]. The use of nonhomogeneity to selectively vary the dose at different sites within the target is another added benefit of hypofractionated radiosurgery as it provides the flexibility to steer a hot spot to the desired target and away from critical structures such as the mandible while treating previously irradiated parotid tumors [11]. In other words, a steeper dose gradient is constructed to answer the clinical need.

Comparative analysis of using daily fractionated SBRT for reirradiation compared to other modalities or regimens is somewhat challenging given the heterogeneity of this patient population. In this study, the reirradiation group comprised patients with very high-risk adverse features. One third of the patients were deemed inoperable, and half either had gross disease or positive margins. With a mean follow-up of 19 months, 44% of the patients failed locally with an actuarial local control rate of 53% at 2 years. This is comparable to other studies using neutron therapy for recurrent salivary gland malignancies, which reported a 5-year local control rate of 26% [8]. Forty-four percent of the patients developed distant failure, and 16% had both local and distant failures [8] consistent with other reports of distant failure representing the dominant pattern of failure [7, 12].

Prognostic analysis revealed, not surprisingly, that presence of gross disease and shorter interval to reirradiation treatment are negative predictors of overall survival on both univariate and multivariate analyses. This is consistent with the results of randomized phase III European multi-institutional trial showing a benefit for reirradiation in the postoperative setting after salvage surgery in patients with high-risk surgical features [13]. On multivariate analysis, there was also a statistically significant correlation between the volume of disease at the time of reirradiation and overall survival. The importance of tumor bulk in predicting survival outcome has also been shown in other studies of reirradiation of head and neck cancers [14, 15].

It had been reported that adjuvant chemotherapy administered concurrently with reirradiation patients improved locoregional control rates as well as distant disease control [8, 16]. A number of phase I and II trials have investigated systemic therapy with mixed results [16]. Recently, concurrent chemoradiation has been shown to improve LRC and PFS in retrospective series [7, 17, 18]. In our series, no statistically significant correlation was seen on either univariate or multivariate analysis perhaps due to the fact that only 39% of the patients received chemotherapy.

It has been established that the higher the dose that can be safely delivered, the higher the probability of disease control [19–21]. Unger et al. have shown that for stereotactic radiosurgery, doses over 30 Gy were associated with improved locoregional outcomes [21]. When we first initiated SBRT reirradiation of salivary gland tumors, we were uncertain about tolerance and late effects for hypofractionated reirradiation. We therefore started cautiously with doses in the 21–25-Gy range. By the time most patients were treated, we had adopted 30–35 Gy for microscopic disease and 35–40 Gy for gross disease with margins as delineated above and with PET/CT and MRI volumes. In our series, a statistically significant correlation was seen between SBRT dose and LRC with doses above 35 Gy yielding better LRC rates. However, as mentioned earlier, the analysis is solely based on four events, which limits the ability to draw any definitive conclusions.

A higher treatment dose was associated with a higher incidence of STN in patients treated with cumulative doses above 90 Gy. Nevertheless, all were successfully salvaged with surgical debridement and reconstruction. It is important to note, however, that since also only four patients experienced toxicity, the data remain inconclusive. In other studies, severe late-term radiation toxicity has been reported on the order of 30% with photon radiotherapy and 69% with neutron radiotherapy [8]. Our finding of statistical correlation between soft tissue necrosis and presence of diabetes is hypothesis generating and may be related to poor wound healing, but warrants further investigation.

## Conclusion

This study has demonstrated the feasibility of using fractionated SBRT reirradiation of recurrent tumors with response rates comparable to other treatment modalities. At early follow-up, treatment was generally well tolerated, but caution needs to be exercised with higher doses to prevent STN. Our retrospective review is, however, limited by potential selection bias, sample size, and heterogeneous patient population and treatment parameters. Future studies with larger sample size, longer follow-up, and less histological variability are warranted.
